# Enhanced Predefined-Time Control for Spacecraft Attitude Tracking: A Dynamic Predictive Approach

**DOI:** 10.3390/s24165127

**Published:** 2024-08-08

**Authors:** Jinhe Yang, Tongjian Guo, Yi Yu, Quanliang Dong, Yifan Jia

**Affiliations:** 1 Changchun Institute of Optics, Fine Mechanics and Physics, Chinese Academy of Sciences, Changchun 130033, China; yangjinhe20@mails.ucas.ac.cn (J.Y.); yuyi@ciomp.ac.cn (Y.Y.); dongquanliang20@mails.ucas.ac.cn (Q.D.); jiayifan20@mails.ucas.ac.cn (Y.J.); 2 University of Chinese Academy of Sciences, Beijing 100049, China

**Keywords:** rigid spacecraft, attitude tracking, predefined-time control, dynamic predictive technology, speediest update predictive period

## Abstract

This study presents a predefined-time control strategy for rigid spacecraft, employing dynamic predictive techniques to achieve robust and precise attitude tracking within predefined time constraints. Advanced predictive algorithms are used to effectively mitigate system uncertainties and environmental disturbances. The main contributions of this work are introducing adaptive global optimization for period updates, which relaxes the original restrictive conditions; ensuring easier parameter adjustments in predefined-time control, providing a nonconservative upper bound on system stability; and developing a continuous, robust control law through terminal sliding mode control and predictive methods. Extensive simulations confirm the control scheme reduces attitude tracking errors to less than 0.01 degrees at steady state, demonstrating the effectiveness of the proposed control strategy.

## 1. Introduction

In the realm of space exploration and the development of space infrastructure, spacecraft are pivotal assets, with the attitude control system (ACS) playing an essential role [1]. The challenge of spacecraft attitude tracking, influenced by unpredictable disturbances and a variety of environmental effects in space, has attracted significant research interest [2].

Recent advancements have seen an increasing focus on fixed-time attitude control for spacecraft. This approach, distinct from finite-time control, provides a predetermined system convergence time [3]. However, the complex relationship between system parameters and fixed-time convergence has remained intricate and ambiguous. To overcome these challenges, the concept of predefined time has been introduced as a conservative estimate for the upper limit of fixed time. This method effectively addresses the limitations of fixed-time control, enhancing the predictability and reliability of system behavior [4].

In response to the operational challenges faced by spacecraft, a range of sophisticated control methods have been developed to meet the stringent mission performance requirements. Optimal control theory has been advanced by Hu et al. [5], and composite adaptive attitude controllers have been innovatively designed by Liu et al. [6]. Lyapunov-based design strategies have been further developed by Hu et al. and Wu et al. [7,8], and robust control along with its integrated applications has been thoroughly explored in several studies [9,10,11]. Additionally, dual-loop controllers incorporating sliding mode control (SMC) and model predictive control (MPC) have proven effective in addressing the complexities of spacecraft attitude tracking [12,13,14].

The exploration of robust control algorithms is critical for mitigating the effects of uncertainty and disturbances encountered by spacecraft in orbit. Among the various robust control methods, sliding mode control (SMC) and its derivatives have shown exceptional capability in managing unknown interferences and uncertainties. Based on this foundation, finite-time sliding mode control methodologies have been developed to ensure the stable convergence of attitude tracking within predefined timeframes, receiving widespread attention for their practical engineering applications [15,16,17]. Adaptive backstepping techniques, combined with second-order sliding mode control laws, have been proposed by Pukdeboon et al. [18] to address finite-time attitude tracking challenges in rigid spacecraft without considering the boundary information about uncertainties and interference. Furthermore, Cao et al. [19] introduced a combination of nonsingular terminal sliding mode control (NTSMC) and the Lyapunov function method to solve the attitude tracking control issues of flexible spacecraft equipped with redundant reaction wheels. Additionally, Pukdeboon et al. [20] proposed a new finite-time attitude tracking controller that uses a finite-time disturbance observer to estimate disturbances under the presence of external interference, verifying that the tracking error converges to zero within a finite time. Moreover, Chen et al. [21] employed a new polynomial finite-time performance function to reduce computational requirements and adopted a simpler error transformation to enhance the speed and accuracy of tracking error convergence. From another standpoint, different types of time-varying sliding mode control were employed to solve the tracking problem of expected attitude trajectories that are known or measurable, and the genetic algorithm optimized the parameters to ensure the strong robustness of the controller [22]. Despite the advances in SMC, there are still notable challenges that need to be addressed. SMC can suffer from chattering effects, which can degrade control performance, making it difficult to ensure fast convergence and high accuracy.

Furthermore, advanced control methods like model predictive control (MPC) have been extensively applied to spacecraft attitude control [14,23,24]. Saki et al. [25] proposed an adaptive soft switching law based on Lyapunov theory to dynamically adjust controller parameters. Multiple optimal sub-models have been utilized, and direct adaptive structure multi-model predictive control has been employed for multi-operation point and maneuvering range attitude tracking. Zhang et al. [26] designed a double-layer nonlinear controller based on the high precision and constraint properties of MPC, ensuring robust system dynamics and resilience to external disturbances. Nonetheless, the inherent uncertainties of these systems pose challenges in obtaining high-precision nominal models, rendering MPC overly conservative regarding disturbance robustness, often based on worst-case scenarios. To address these issues, an ideal Generalized Predictive Control (GPC) framework has been employed, which accommodates unknown terms and ensures the optimization of closed-loop system performance while maintaining the efficiency and accuracy of the control method, making it particularly suitable for analyzing trajectory tracking control problems amid parameter uncertainty and unknown constraint interference [27,28,29]. Predictive control methods often require precise system models and can be computationally intensive, making real-time implementation challenging.

To overcome these challenges, this paper introduces a novel predefined-time attitude control scheme for rigid spacecraft, designed to achieve accurate attitude tracking within a predefined timeframe using quaternion representations for spacecraft attitude. The scheme leverages dynamic prediction techniques and the fastest optimizing prediction period to enhance control robustness against parameter uncertainties and disturbances. The main contributions of this study include:

1. Adaptive Global Optimization for Period Updates: The introduction of new theorems allows for the use of an adaptive global optimization approach to achieve period updates, relaxing the original restrictive conditions in control schemes proposed by Sun G et al. [30].
2. Ease of Parameter Adjustment in Predefined-Time Control: Compared to fixed-time control methods, the parameters related to predefined-time control are more easily adjustable, providing a nonconservative upper bound on system stability. This makes the determination of stabilization time for the closed-loop system more straightforward and practical.
3. Continuous and Robust Control via Terminal Sliding Mode Control and Predictive Methods: This work diverges from the approach of Chen et al. [31], which employs terminal sliding mode control technology to design predefined-time attitude controllers. Instead, it utilizes a method of optimizing prediction periods and predictive control to derive the control law, ensuring that the proposed control scheme is both continuous and robust.

For the sake of clarity and to facilitate subsequent mathematical expressions, we introduce the following notations in advance:

- The symbols *R*, $R^{+}$, *N*, $N^{+}$, and $R^{n}$ denote the set of real numbers, the set of positive real numbers, the set of natural numbers, the set of positive integers, and the *n*-dimensional real number space, respectively.
- For any matrix $A\in R^{n\times n}$, $\lambda_{\max}\left(A\right)$ and $\lambda_{\min}\left(A\right)$ represent the maximum and minimum eigenvalues of *A*, respectively.
- For any vector $x={\left[x_{1},x_{2},\ldots ,x_{n}\right]}^{T}\in R^{n}$, ∥x∥ denotes the Euclidean norm of *x*.
- For any vector $a={\left[a_{1},a_{2},a_{3}\right]}^{T}\in R^{3}$, the notation $a^{\times }$ denotes the corresponding skew-symmetric matrix:
  $$a^{\times }=\left[\begin{array}{ccc} 0 & -a_{3} & a_{2}\\ a_{3} & 0 & -a_{1}\\ -a_{2} & a_{1} & 0 \end{array}\right].$$
- For any vector $a={\left[a_{1},a_{2},a_{3}\right]}^{T}\in R^{3}$ and any positive scalar γ, $sig^{\gamma }\left(a\right)$ denotes the vector:
  $$sig^{\gamma }\left(a\right)=\left[\right|a_{1}{|}^{\gamma }sign\left(a_{1}\right),|a_{2}{|}^{\gamma }sign\left(a_{2}\right),|a_{3}{{|}^{\gamma }sign\left(a_{3}\right)]}^{T},$$
  where $sign\left(a_{i}\right)$, for i=1,2,3, is defined as:
  $$sign\left(a_{i}\right)=\left\{\begin{array}{cc} \frac{a_{i}}{|a_{i}|} & \mathrm{if}\;a_{i}\neq 0\\ 0 & \mathrm{if}\;a_{i}=0 \end{array}\right..$$
- The symbol $C^{i}\left(i\in N\right)$ denotes the set of all differentiable functions whose *i*-th derivative is continuous. If a function $f:R^{n}\to R$ has partial derivatives and is continuous up to the *k*-th order, 1≤k≤∞, then the function *f* is called a $C^{k}$ function. Here, $C^{0}$ represents continuous functions, and $C^{\infty }$ represents smooth functions, i.e., functions that have continuous partial derivatives of any order.

## 2. Spacecraft Attitude Control Problem

**Figure 1:**
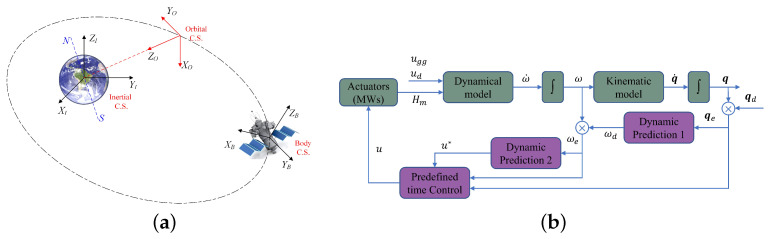
(**a**) Illustration of spacecraft orbital motion and coordinate system; (**b**) schematic diagram of the designed controller structure.

As is well known, Euler angles are frequently used to parameterize spacecraft attitude due to their clear and straightforward physical concept. However, Euler angles may encounter singularity or gimbal lock issues when describing the parameterized attitude of a spacecraft. To address these challenges, this paper adopts the quaternion representation for spacecraft attitude. The spacecraft, modeled as a rigid body, operates in low Earth orbit as depicted in Figure 1a. Its actuators are momentum wheels (MWs), which provide torque about three orthogonal axes defined with respect to the body coordinate system *B*.

In the spacecraft attitude control system, when MWs are used as actuators, they contribute additional angular momentum, denoted as $H_{m}=J_{m}\omega_{m}$, where $J_{m}$ is the inertia matrix and $\omega_{m}$ is the angular velocity of the MWs. Furthermore, the kinematics and dynamics of the spacecraft are governed by the following nonlinear equations [14,32]:

(1) $$\begin{array}{c} {\dot{q}}_{v}=P\left(q\right)\omega ,{\dot{q}}_{4}=-\frac{1}{2}q_{v}^{T}\omega \end{array}$$

(2) [JJm]ω˙ω˙m+ω×[JJm]ωωm=ud+ugg

where $q={\left[q_{v}^{T},q_{4}\right]}^{T}\in R^{4}$ represents the quaternion describing the orientation of the spacecraft body frame *B* relative to the inertial coordinate system I. The vector $q_{v}^{T}={\left[q_{1},q_{2},q_{3}\right]}^{T}\in R^{3}$ and the scalar $q_{4}\in R$ ensure $q^{T}q=1$. The angular velocity $\omega ={\left[\omega_{x},\omega_{y},\omega_{z}\right]}^{T}\in R^{3}$ is defined in the body frame *B*. The inertia matrix of the spacecraft is denoted by $J=J^{T}\in R^{3\times 3}$, and $P\left(q\right)\in R^{3\times 3}$ is defined as $P\left(q\right)=\frac{1}{2}\left[q_{v}^{\times }+q_{4}I_{3}\right]$.

The torque applied to the system, due to the orbital gravity gradient and unknown, bounded disturbances, is represented by $u_{d}\in R^{3}$ and $u_{gg}\in R^{3}$, respectively. Here, $u_{gg}=3\omega_{0}^{2}\left(k_{o}\times Jk_{o}\right)$, where $\omega_{0}=\sqrt{\mu /r_{c}^{3}}$ denotes the orbital velocity of a spacecraft in a near-Earth orbit, $\mu =398,600\;{\mathrm{km}}^{3}/s^{2}$ represents the gravitational constant of the Earth, $r_{c}$ is the distance between the spacecraft and the Earth’s center of mass, and $k_{o}$ denotes the third column of the direction cosine matrix of the spacecraft [15].

Based on the nonlinear dynamics model of the rigid spacecraft’s attitude given by (1) and (2), and considering the actual control process of the spacecraft, the following assumptions can be made.

**Assumption** **1.** *We assume that the form of the inertia matrix J in (2) is $J=J_{0}+\Delta J$, where $J_{0}$ and ΔJ represent the known nominal matrix and uncertain part of J, and ΔJ satisfies $∥\Delta J∥\leq J_{M}$, where $J_{M}$ is a known positive constant.*

**Assumption** **2.** *We assume that the disturbance $u_{d}$ is differentiable and bounded, i.e., $∥u_{d}∥\leq u_{M}$, where $u_{M}$ is a known positive constant.*

**Assumption** **3.** *We assume that the system input u is bounded.*

In this study, the desired spacecraft attitude relative to the inertial coordinate system I is represented by the quaternion $q_{d}={\left[q_{dv}^{T},q_{d4}\right]}^{T}\in R^{4}$, and the desired attitude angular velocity by $\omega_{d}={\left[\omega_{dx},\omega_{dy},\omega_{dz}\right]}^{T}\in R^{3}$. The attitude tracking error between the desired and actual attitudes is denoted as $q_{e}={\left[q_{ev}^{T},q_{e4}\right]}^{T}\in R^{4}$, with components $q_{ev}={\left[q_{e1},q_{e2},q_{e3}\right]}^{T}\in R^{3}$ satisfying the norm constraint $q_{ev}^{T}q_{ev}+q_{e4}^{2}=1$.

The error quaternion $q_{e}$ is defined by the equation:

(3) $$\begin{array}{c} q_{ev}=q_{d4}q_{v}-q_{dv}^{\times }q_{v}-q_{4}q_{dv},q_{e4}=q_{dv}^{T}q_{v}+q_{d4}q_{4} \end{array}$$

To aid in the controller design, it is assumed that both $\omega_{d}$ and ${\dot{\omega }}_{d}$ are bounded. The angular velocity error $\omega_{e}$ is defined as $\omega -C_{e}\omega_{d}$, where the matrix $C_{e}$ is determined from the tracking error and is expressed as $C_{e}=\left(q_{e4}^{2}-q_{ev}^{T}q_{ev}\right)I_{3}+2q_{ev}q_{ev}^{T}-2q_{e4}q_{ev}^{\times }$. In addition, $C_{e}$ satisfies ${\dot{C}}_{e}=-\omega_{e}^{\times }C_{e}$. The kinematic equation governing the attitude tracking error $q_{e}$ is given by:

(4) $$\begin{array}{c} {\dot{q}}_{ev}=P\left(q_{e}\right)\omega_{e},{\dot{q}}_{e4}=-\frac{1}{2}q_{ev}^{T}\omega_{e} \end{array}$$

Here, $P(q_{e})$ impacts the dynamics of $\omega_{e}$, influencing the attitude control process.

Combining (2) and (4), the differential control equation for the attitude tracking angular velocity error $\omega_{e}$ is as follows:

(5) $$\begin{array}{c} J_{0}{\dot{\omega }}_{e}=J_{0}\omega_{e}^{\times }C_{e}\omega_{d}-J_{0}C_{e}\omega_{d}-\omega^{\times }J_{0}\omega +u+d \end{array}$$

where $u=-J_{m}{\dot{\omega }}_{m}-\omega^{\times }J_{m}\omega_{m}+u_{gg}$, $d=u_{d}-\Delta J\omega_{e}^{\times }C_{e}\omega_{d}+{\left(C_{e}\omega \right)}^{\times }\Delta J\omega +\omega_{e}^{\times }\Delta J\omega$.

The upper bound of the uncertain/disturbance part of the system can be determined by the following expression:

(6) $$\begin{array}{c} ∥d∥\leq b_{0}+b_{1}∥\omega ∥+b_{2}{∥\omega ∥}^{2} \end{array}$$

in which $b_{0}$, $b_{1}$, and $b_{2}$ are unknown positive constants, which satisfy that $b_{0}$ is strictly greater than the constant term of $∥d∥$, $b_{1}$ is strictly greater than the coefficient of the term ∥ω∥ of $∥d∥$, and $b_{2}$ is strictly greater than the coefficient of the term ${∥\omega ∥}^{2}$ of $∥d∥$.

The primary objective of this study is to design a robust control law $u\in R^{3}$ (as illustrated in Figure 1b), ensuring that both the attitude tracking error $q_{ev}$ and the angular velocity error $\omega_{e}$ converge asymptotically to zero under disturbance-free conditions, or within a small bounded region when subject to external disturbances. Critically, the predefined stabilization time is designed to be invariant with respect to the initial system states, affirming the control’s robustness across varying operational scenarios.

Before proceeding to the main results of this paper, some preliminaries need to be presented.

## 3. Preliminaries

Introduction to the following definitions and mathematical lemmas can be crucial for understanding the main results in this article.

Consider the following nonlinear system:

(7) $$\begin{array}{c} \dot{x}=f\left(x,\theta ,d\right),x\left(0\right)=x_{0} \end{array}$$

where $x\in R^{n}$ is the state vector of the system; $\theta \in R^{b}$ is the system parameter of the system (7); $d\in R^{n}$ is a vector of unknown disturbances and dynamic uncertainties; $x_{0}$ is the initial state; and $f:R^{n}\to R$ is a smooth nonlinear function and its origin is assumed to be the equilibrium point of the system, i.e., f(0,θ)=0.

**Definition** **1** (Finite-time stability [33]). *The system (7) is considered globally finite-time stable if it is globally asymptotically stable, and any solution reaches equilibrium within a finite time, i.e.,*

(8) $$\begin{array}{c} \forall t\geq T\left(x_{0}\right):x\left(t,x_{0}\right)=0 \end{array}$$

*where $T(x_{0})$ represents the actual convergence time of the system.*

**Definition** **2** (Fixed-time stability [34]). *If the system (7) satisfies the following two conditions, it is referred to as a globally fixed-time stable system: (i) it can reach a stable state within a finite time, and (ii) the stable time $T(x_{0})$ is globally bounded and independent of the initial state of the system, i.e.,*

(9) $$\begin{array}{c} \exists T_{\max}>0:\forall x_{0}\in R^{n},T\left(x_{0}\right)\leq T_{\max} \end{array}$$

*where $T_{\max}$ is an estimate of the convergence time of the system.*

**Definition** **3** (Predefined-time stability [35]). *For system parameter **θ** and a predetermined parameter $T_{c}=T\left(\theta \right)>0$ (where $T_{c}$ is an adjustable parameter), if the system (7) is fixed-time stable and the stable time $T:R^{n}\to R$ satisfies*

(10) $$\begin{array}{c} T\left(x_{0}\right)\leq T_{c},\forall x_{0}\in R^{n} \end{array}$$

*then the system is referred to as a globally predefined-time stable system. Furthermore, if the minimum bound of the predefined-time function $T_{f}$ satisfies $T_{f}=\sup_{x_{0}\in R}T\left(x_{0}\right)=T_{c}$, then $T_{c}$ is referred to as a strong predefined time; otherwise, $T_{c}$ is referred to as a weak predefined time.*

**Lemma** **1.** *Assuming that $V\left(x\right):R^{n}\to R^{+}\cup \left\{0\right\}$ is a continuous positive definite radial unbounded function that satisfies the following: (i) V(x)=0⇒x∈M, where $M\in R^{n}$ is a nonempty set; (ii) for any $V\left(x\right)>0$, there exist parameters α, β, k, p, q>0, and 0<kp<1<kq such that*

(11) $$\begin{array}{c} \dot{V}\leq -\frac{C_{v}}{T_{c}}{\left(\alpha V^{p}+\beta V^{q}\right)}^{k} \end{array}$$

*and $\forall t>T_{c}$, it can be guaranteed that $V\left(x\right)=0$ and system (7) converges within the predefined time $T_{c}$, where $T_{c}$ is the convergence time.*

*If the parameter vector is defined as $\theta ={\left[\alpha ,\beta ,p,q,k\right]}^{T}\in R^{5}$, then the predefined-time function satisfies $T_{f}=C_{v}\left(\theta \right)$, where $C_{v}\left(\theta \right)$ can be calculated by the following expression [36]:*

(12) $$\begin{array}{c} C_{v}\left(\theta \right)=\frac{\Gamma \left(m_{p}\right)\Gamma \left(m_{p}\right)}{\alpha^{k}\Gamma \left(k\right)\left(q-p\right)}{\left(\frac{\alpha }{\beta }\right)}^{m_{p}} \end{array}$$

*where $m_{p}=\frac{1-kp}{q-p}$, $m_{q}=\frac{kq-1}{q-p}\in N^{+}$; $\Gamma \left(\cdot \right)$ is the gamma function that satisfies $\Gamma \left(z\right)=\int_{0}^{+\infty }e^{-t}t^{z-1}dt$.*

**Lemma** **2.** *For any $x_{i}\in R^{+}\left(i=1,2,\cdots ,n\right)$, and a real number $p\in \left(0,1\right]$, then [37]*

(13) $$\begin{array}{c} {\left(\sum_{i=1}^{n}\left|x_{i}\right|\right)}^{p}\leq \sum_{i=1}^{n}{\left|x_{i}\right|}^{p}\leq n^{1-p}{\left(\sum_{i=1}^{n}\left|x_{i}\right|\right)}^{p} \end{array}$$

**Lemma** **3.** *For any $x_{i}\in R^{+}\left(i=1,2,\cdots ,n\right)$, and a real number q>1, then [37]*

(14) $$\begin{array}{c} \sum_{i=1}^{n}{\left|x_{i}\right|}^{q}\leq {\left(\sum_{i=1}^{n}\left|x_{i}\right|\right)}^{q}\leq n^{q-1}\sum_{i=1}^{n}{\left|x_{i}\right|}^{q} \end{array}$$

**Lemma** **4.** *For any real-valued continuous function $f\left(x_{1},\cdots ,x_{i},\cdots ,x_{n}\right)\in C\left(R^{n},R^{n}\right)$, where $i\in N_{1:n}$, there exist i smooth scalar functions $f_{i}\left(x_{i}\right)\geq 1$, such that $|f\left(x_{1},\cdots ,x_{i},\cdots ,x_{n}\right)|\leq \prod f_{i}\left(x_{i}\right)$ [38].*

**Lemma** **5.** *If $\lim_{t\to \infty }f\left(t\right)<\infty$ exists, and $\dot{f}$ is uniformly continuous (or $\ddot{f}$ is bounded), then $\lim_{t\to \infty }\dot{f}\left(t\right)=0$.*

## 4. Main Results

In this section, we propose a novel predetermined time attitude tracking control scheme for rigid spacecraft. To facilitate the controller design, we introduce a sliding mode variable:

$$\begin{array}{c} s=\omega_{e}+sig^{\frac{1}{2}}\left(\vartheta \right) \end{array}$$

where the auxiliary variable $\vartheta =sig^{2}\left(\omega_{e}\right)+\frac{C_{v}^{2}}{2T_{c1}^{2}}\left(\alpha sig^{p}\left(q_{ev}\right)+\beta sig^{q}\left(q_{ev}\right)\right)$ and relevant parameters are defined according to Lemma 1. The controller development process is based on dynamic prediction techniques, and can be explained as follows.

Step 1. Dynamic prediction model for the system input-output.

Design the controller using a general form of optimization method to achieve asymptotic stability of the closed-loop system. Ensure that the spacecraft output $y={\left[q_{ev},\omega_{e}\right]}^{T}\in R^{6}$ of the lower-triangular system converges to the origin in an optimal way according to the following performance index:

(15) $$\begin{array}{c} J=\frac{1}{2}\int_{0}^{T}y{\left(t+\tau \right)}^{2}d\tau \end{array}$$

where T>0 is the prediction period.

**Figure 2:**
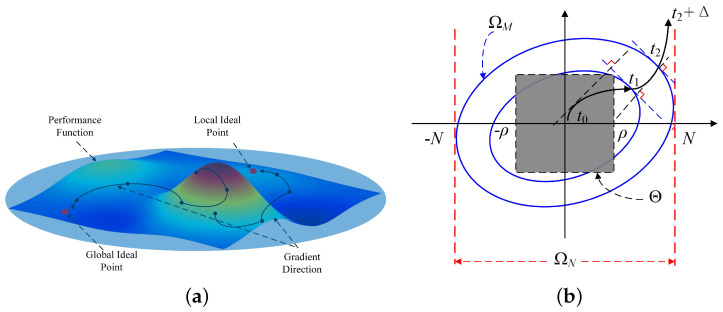
A sketch description of the predefined−time convergence of the steepest descent update rule. (**a**) The direction of the steepest descent update vector (The equipotential surface contour map represents the vector.); (**b**) a sketch of the predefined−time convergence.

The above problem will be solved by a variable prediction period $T=T\left(0\right)e^{-L}$, where $T\left(0\right)>0$, and the update formula for *L* (as shown in Figure 2a) is given by:

(16) $$\begin{array}{cc} \Delta L= & \frac{\nu }{1-\beta }-\beta \frac{\partial J(L,x)}{\partial L}\\ \beta = & {\mathrm{argmin}}_{\beta }f\left(\nu -\beta \nabla J\right)=0 \end{array}$$

where β is the update learning rate. The steepest descent update rule is transformed into a hidden Lipschitz nonlinear hybrid optimization form, ensuring that the control process achieves global optimization rather than merely local improvements. This transformation is critical for enhancing the robustness and effectiveness of the control strategy across all operational regimes. The optimization approach is equivalent to the adaptive change rate (17), which dynamically adjusts based on the evolving system conditions, further ensuring the comprehensive applicability and efficiency of the control method [39,40,41].

(17) $$\begin{array}{c} \dot{L}=f\left(L,\dot{\hat{v}},x\right),L\left(0\right)=0,f:R^{+}\times R\to R^{+} \end{array}$$

Based on the configuration of system (15), the future output $\hat{y}\left(t+\tau \right)$ within the prediction horizon (i.e., 0<τ<T) is predicted using the following Taylor series expansion:

(18) $$\begin{array}{cc} \hat{y}\left(t+\tau \right) & =\alpha_{0}x_{1}+\alpha_{1}\tau x_{2}+\cdots +\alpha_{n-1}\frac{\tau_{n-1}}{(n-1)!}x_{n}\\ \; & +\alpha_{n}\left[\frac{\tau^{n}}{n!}u+\cdots +\frac{\tau^{n+r}}{(n+r)!}u^{\left(r\right)}\right]\\ \; & =\bar{\Gamma }x+\tilde{\Gamma }U \end{array}$$

where $\bar{\Gamma }≜\left[\alpha_{0},\cdots ,\alpha_{n-1}\frac{\tau^{n-1}}{(n-1)!}\right]$, $\tilde{\Gamma }≜\alpha_{n}\left[\frac{\tau^{n}}{n!},\cdots ,\frac{\tau^{n+r}}{(n+r)!}\right]$, $U≜{\left[u,\cdots ,u^{\left(r\right)}\right]}^{T}$, r∈N denotes the control sequence, and $\alpha_{i}$ is the defined constants related to the kinematic-dynamic properties of the spacecraft.

By the form of (18), the performance index for prediction (15) can be obtained in the following form:

(19) $$\begin{array}{c} \hat{J}=\frac{1}{2}\int_{0}^{T}\hat{y}{\left(t+\tau \right)}^{2}d\tau =\frac{1}{2}x^{T}\Gamma_{1}x+x^{T}\Gamma_{2}U+\frac{1}{2}U^{T}\Gamma_{3}U \end{array}$$

where $\Gamma_{1}=\int_{0}^{T}{\bar{\Gamma }}^{T}\bar{\Gamma }d\tau \in R^{n\times n}$, $\Gamma_{2}=\int_{0}^{T}{\bar{\Gamma }}^{T}\tilde{\Gamma }d\tau \in R^{n\times (r+1)}$, $\Gamma_{3}=\int_{0}^{T}{\tilde{\Gamma }}^{T}\tilde{\Gamma }d\tau \in R^{\left(r+1)\times (r+1\right)}$.

Taking the partial derivative of $\hat{J}$ with respect to *U* yields $\partial \hat{J}/\partial U=\Gamma_{2}^{T}x+\Gamma_{3}U$. In this paper, $\Gamma_{3}$ is a positive definite matrix. By setting $\partial \hat{J}/\partial U=0$ and ensuring that $\partial^{2}\hat{J}/\partial U^{2}>0$, the optimal control sequence is obtained as $U^{*}=-\Gamma_{3}^{-1}\Gamma_{2}^{T}x$.

Assuming that $\Gamma_{2}\left(i,j\right)=p_{i,j}T^{n+i+j-1}$ and $\Gamma_{3}\left(i,j\right)=q_{i,j}T^{2*n+i+j-1}$ have such forms, the simplified form of the control law can be obtained as follows:

(20) $$\begin{array}{c} U^{*}=-e^{L}\frac{\kappa_{1}}{T^{n}}x_{1}-e^{L}\frac{\kappa_{2}}{T^{n-1}}x_{2}-\cdots -e^{L}\frac{\kappa_{n}}{T}x_{n} \end{array}$$

where $p_{i,j}$, $q_{i,j}$ and $\kappa_{i}$ are constants that depend only on *r* and *n*. The above derivation still holds when the prediction horizon is variable or state dependent [42].

For the convenience of the proof of the predictive control described in this paper, let $z_{i}≜x_{i}e^{-L}\left(i\in N_{1:n}\right)$, and the system (15)–(20) can be constructed into a more compact expression as follows:

(21) $$\begin{array}{c} \dot{z}=-I\dot{L}z+Az+e^{-L}\Phi \left(\theta ,x\right) \end{array}$$

where *I* denotes the identity matrix, $z≜{\left[z_{1},z_{2},...,z_{n}\right]}^{T}$, $\Phi \left(\theta ,x\right)≜{\left[\varphi_{1},\varphi_{2},\cdots ,\varphi_{n}\right]}^{T}$, $A≜\left[\begin{array}{cccc} 0 & \alpha_{1} & \cdots & 0\\ \vdots & \vdots & \ddots & 0\\ 0 & 0 & \cdots & \alpha_{n-1}\\ e^{L}\frac{\kappa_{1}}{T^{n}\left(0\right)} & e^{L}\frac{\kappa_{2}}{T^{n-1}\left(0\right)} & \cdots & e^{L}\frac{\kappa_{n}}{T(0)} \end{array}\right]$.

According to the relevant theory of optimal control [40,41], the stability of the nominal system (19) depends only on the control order *r*. By choosing the control order *r* appropriately, there exists a positive definite and symmetric matrix $P\in R^{n\times n}$ such that $A^{T}P+PA=-I$. In this paper, the update of the prediction horizon is presented in the following adaptive form:

(22) $$\begin{array}{c} \left.\left\{\begin{array}{cc} \dot{\hat{v}} & =\frac{\rho_{1}}{\gamma }e^{-L}{∥z∥}^{2}\\ \dot{L} & =\frac{1}{\varepsilon_{1}}\max\left(0,\rho_{2}-\varepsilon_{2}e^{L}\frac{\kappa }{T(0)}\right),L\left(0\right)=0 \end{array}\right.\right. \end{array}$$

where $\rho_{1}$ and $\rho_{2}$ are adjustable parameters that satisfy $\frac{1}{\sqrt{n}K\lambda_{\max}\left(P\right)}>\rho_{1}>0$, $\rho_{2}>0$. The speediest update method can be shown to be consistent with adaptive characteristics through (22).

Considering the computational complexity limitations of the dynamics predictive controller for rigid spacecraft, the optimization order n=1 is set as proposed in (20). Higher-order residuals and remaining parameters are adjusted using the adaptive parameter adjustment described in Equation (22). Consequently, the following can be derived:

(23) $$\begin{array}{cc} \omega_{d}\left(t\right) & =P^{-1}\left(q_{e}\right)\left(-\frac{\kappa_{1}}{T_{1}}I_{3}q_{ev}+{\dot{q}}_{dv}+{\hat{v}}_{1}\right)\\ u^{*}\left(t\right) & =J_{0}\left(-\frac{\kappa_{2}}{T_{2}}I_{3}\omega_{e}+J_{0}^{-1}\omega^{\times }J_{0}\omega +{\hat{v}}_{2}\right) \end{array}$$

where $T_{1}=T_{1}\left(0\right)e^{-L_{1}}$, $T_{2}=T_{2}\left(0\right)e^{-L_{2}}$ and $\kappa_{1}=\kappa_{2}=4$.

The ${\hat{v}}_{i}$ represent the unknown estimation laws defined in (22), satisfying the condition that ${\hat{v}}_{i}\in R^{3}\left(i=1,2\right)$.

**Theorem** **1.** *For the attitude control system (4) and (5) of a rigid spacecraft, the closed-loop system is globally asymptotically stable under dynamic predictive control in the form of (23). The dynamic prediction period is determined by (22), and thus, the spacecraft’s attitudes error $q_{ev}\to 0$ and attitude angular velocity error $\omega_{e}\to 0$ exist.*

**Proof.**  The closed-loop system discussed in this proof section satisfies the following conditions:

1. It is evident that all states in the closed-loop system are uniformly bounded and that the system state converges to the origin, i.e., $\lim_{t\to \infty }x=0$.
2. According to Lemma 3, for each $C^{0}$ function $\partial \varphi_{i}/\partial x_{j},i\in N_{1:n},j\in N_{1:i}$, there exist smooth scalar functions $a_{i,j}\left(\theta \right)\geq 1$ and $b_{i,j}\left({\bar{x}}_{i}\right)\geq 1$ such that $|\partial \varphi_{i}/\partial x_{j}|\leq a_{i,j}\left(\theta \right)b_{i,j}\left({\bar{x}}_{i}\right)$.

Considering that *v* is an unknown variable that depends on θ, which needs to be evaluated in the controller (as shown in (23)), the following definition is provided for the convenience of the following proof.

Let $v≜\max_{i\in N_{1:n}}\left\{a_{i,1}\left(\theta \right),a_{i,1}\left(\theta \right),\cdots ,a_{i,1}\left(\theta \right)\right\}$ and we have $\left[\underline{v},\bar{v}\right]$, where $\underline{v}$ and $\bar{v}$ are known constants. We define $\Omega_{M}≜\left\{{\left(z^{T},\tilde{v}\right)}^{T}\in R^{n+1}|V\left(z,\tilde{v}\right)\leq M\right\}$, $\Omega_{N}≜{\left[-N,N\right]}^{n}$, where $M≜\max_{z\in {\left[-\rho ,\rho \right]}^{n},\hat{v}\in \left[v-\rho ,\bar{v}+\rho \right]}V\left(z,\tilde{v}\right)$, $N≜\max_{{\left(z^{T},\hat{v}\right)}^{T}\in \Omega_{M}}{∥z∥}_{\infty }$ [42].

The domain of the closed-loop system under consideration in this proof section is defined as ${\left(x{\left(0\right)}^{T},\hat{v}{\left(0\right)}^{T}\right)}^{T}\in \Theta ≜{\left[-\rho ,\rho \right]}^{n+1}$, where ρ is a positive constant for any initial state that satisfies the following domain condition: ${\left(x{\left(0\right)}^{T},\hat{v}{\left(0\right)}^{T}\right)}^{T}\in \Theta \Rightarrow \tilde{v}\left(0\right)\in \left[\underline{v}-\rho ,\bar{v}+\rho \right]$.

We construct a Lyapunov function as $V\left(z,\tilde{v}\right)≜W\left(z\right)+\gamma {\tilde{v}}^{T}\tilde{v}$, where $W\left(z\right)=z^{T}Pz$, $\tilde{v}=v-\hat{v}$ is an estimate of *v*, and γ>0 is a design parameter. Based on the above definitions, we obtain the following constraint: $\forall {\left(z^{T},\tilde{v}\right)}^{T}\in \Omega_{M}$, $\exists \varepsilon_{2}\in \left[0,1\right]$, s.t.$\dot{V}\left(z,\tilde{v}\right)\leq -e^{L}\frac{\kappa }{T(0)}\left(1-\varepsilon_{2}\right){∥z∥}^{2}$.

We can differentiate W(z) with respect to the form of system (21):

(24) $$\begin{array}{c} \dot{W}\left(z\right)=-e^{L}\frac{\kappa }{T(0)}{∥z∥}^{2}+e^{-L}z^{T}P\Phi -\dot{L}z^{T}Pz \end{array}$$

First, we estimate the last two terms on the right side of (24).

When $x_{i}\neq 0,i\in N_{1:n}$ holds, by the mean value lemma, we have:

(25) $$\begin{array}{cc} |\psi_{i}\left(\theta ,{\bar{x}}_{i}\right)| & \leq e^{-L}\left|\psi_{i}\left(\theta ,\bar{x_{i}}\right)-\psi_{i}\left(\theta ,0\right)\right|\\ \; & =e^{-L}\left|x_{1}\frac{\partial \psi_{i}}{\partial x_{1}}{{|}_{x_{1}=\xi_{1},\cdots ,x_{i}=\xi_{i}}+x_{i}\frac{\partial \psi_{i}}{\partial x_{i}}|}_{x_{1}=\xi_{1},\cdots ,x_{i}=\xi_{i}}\right|\\ \; & \leq e^{-L}\tilde{v}\left(\right|x_{1}\left|+\right|x_{2}\left|+\cdots +\right|x_{i}\left|\right)\cdot \max\left\{b_{i,1}\left({\bar{\xi }}_{i}\right),b_{i,2}\left({\bar{\xi }}_{i}\right),\cdots ,b_{i,i}\left({\bar{\xi }}_{i}\right)\right\} \end{array}$$

Under the condition that $x\in \Omega_{N}$, where in $\xi_{i}\in \left(0,x_{i}\right)$. Thus, we can obtain:

(26) $$\begin{array}{cc} |\psi_{i}\left(\theta ,{\bar{x}}_{i}\right)e^{-L}| & \leq e^{-L}K(|x_{1}\left|+\right|x_{2}\left|+\cdots +\right|x_{i}\left|\right)\\ \; & =\tilde{v}K(e^{-L}|x_{1}|+e^{-L}|x_{2}|+\cdots +e^{-L}|x_{i}\left|\right)\\ \; & =\tilde{v}K(|z_{1}\left|+\right|z_{2}\left|+\cdots +\right|z_{i}\left|\right)\\ \; & \leq \tilde{v}K(|z_{1}\left|+\cdots +\right|z_{i}\left|+\cdots +\right|z_{n}\left|\right)\\ \; & \leq \sqrt{n}K∥z∥\tilde{v} \end{array}$$

where $K=\max_{i\in N_{1:n},x\in \Omega_{N}}\left\{b_{i,1}\left({\bar{\xi }}_{i}\right),b_{i,2}\left({\bar{\xi }}_{i}\right),\cdots ,b_{i,i}\left({\bar{\xi }}_{i}\right)\right\}\geq 1$ is a known constant. Thus, we can obtain:

(27) $$\begin{array}{c} \left|z^{T}P\Phi \right|\leq \sqrt{n}K{∥z∥}^{2}{\tilde{v}}^{T}\lambda_{\max}\left(P\right) \end{array}$$

By constructing the proof through the selection of adjustable parameters that satisfy the Hurwitz condition, it can be shown that *P* is a positive definite matrix, which yields the following result:

(28) $$\begin{array}{c} 2z^{T}Pz\geq \varepsilon_{1}{∥z∥}^{2}\geq 0 \end{array}$$

where $\varepsilon_{1}\in \left(0,2\lambda_{\min}\left(P\right)\right)$.

Combining (26)–(28), we can transform (24) to:

(29) $$\begin{array}{c} \dot{W}\left(z\right){|}_{x\in \Omega_{N}}\leq -e^{L}\frac{\kappa }{T(0)}{∥z∥}^{2}+e^{-L}\sqrt{n}{K∥z∥}^{2}{\tilde{v}}^{T}\lambda_{\max}\left(P\right)-\varepsilon_{1}\dot{L}{∥z∥}^{2} \end{array}$$

Furthermore, it can be derived that:

(30) $$\begin{array}{cc} \dot{V}\left(z,\tilde{v}\right){|}_{x\in \Omega_{N}} & =\dot{W}-\gamma {\tilde{v}}^{T}\dot{\hat{v}}\leq -e^{L}\frac{\kappa }{T(0)}{∥z∥}^{2}+e^{-L}\sqrt{n}K\tilde{v}\lambda_{\max}\left(P\right){∥z∥}^{2}-\varepsilon_{1}\dot{L}{∥z∥}^{2}-\gamma {\tilde{v}}^{T}\dot{\hat{v}}\\ \; & \leq -e^{L}\frac{\kappa }{T(0)}\left(1-\varepsilon_{2}\right){∥z∥}^{2}-\left(\varepsilon_{2}e^{L}\frac{\kappa }{T(0)}+\varepsilon_{1}\dot{L}\right){∥z∥}^{2}\\ \; & +{\tilde{v}}^{T}(e^{-L}\sqrt{n}K\lambda_{\max}\left(P\right){∥z∥}^{2}-\gamma \dot{\hat{v}})\\ \; & =-e^{L}\frac{\kappa }{T(0)}\left(1-\varepsilon_{2}\right){∥z∥}^{2}-\left(\varepsilon_{2}e^{L}\frac{\kappa }{T(0)}+\varepsilon_{1}\dot{L}\right){∥z∥}^{2}+{\tilde{v}}^{T}(e^{-L}\rho_{1}{∥z∥}^{2}-\gamma \dot{\hat{v}}) \end{array}$$

Let $\varepsilon_{2}$ and $\rho_{1}=\sqrt{n}K\lambda_{\max}\left(P\right)\in \left(0,1\right)$. Taking (23) into (30) yields that domain condition holds.

For the convenience of the proof, suppose that $V\left(t\right)≜V\left(z,\tilde{v}\right)$. When L(0)=0, there exists ${\left(x{\left(0\right)}^{T},\hat{v}{\left(0\right)}^{T}\right)}^{T}\in \Omega_{M}$ with x(0)≠0 such that V(0)≤M and $\dot{V}\left(0\right)<0$ (if x≡0, t≥0 must be ensured). To simplify the subsequent proof, we assume that the Lyapunov function has the form ${∥z∥}^{2}\geq {∥x∥}^{2}e^{-2L}$ and satisfies $\dot{V}{|}_{x\in \Omega_{N}}\leq -e^{-L}\frac{\kappa }{T(0)}\left(1-\varepsilon_{2}\right){∥x∥}^{2}$.

Through the aforementioned simplification, the form of the domain is defined as ${\left(x^{T},{\hat{v}}^{T}\right)}^{T}\in \Omega_{M}$, $\forall {\left(x{\left(0\right)}^{T},\hat{v}{\left(0\right)}^{T}\right)}^{T}\in \Theta$. There exists $t_{2}\geq 0$ such that the Lyapunov function satisfies $\dot{V}\left(0\right)<0$. Hence, the following relational equation necessarily exists as follows: V<M holds for $t\in \left(0,t_{2}\right)$; $V\left(t_{2}\right)=M$; V>M holds for $t\in \left(t_{2},t_{2}+\Delta \right)$ where Δ>0 (as shown in Figure 2b).

Therefore, we can conclude that:

(31) $$\begin{array}{cc} V\left(t_{2}\right)-V\left(0\right) & =\int_{0}^{t_{2}}\dot{V}\left(t\right)dt\\ \; & \leq -\left(1-\varepsilon_{2}\right)\int_{0}^{t_{1}}e^{-L}\frac{\kappa }{T(0)}{∥x∥}^{2}dt\leq 0 \end{array}$$

As $V\left(t_{2}\right)\geq V\left(0\right)$, we have $\int_{0}^{t_{2}}{∥x∥}^{2}e^{-L}\frac{\kappa }{T(0)}dt=0\Leftrightarrow x\equiv 0,t\in \left(0,t_{2}\right)$. In fact, *z*, γ, and *L* are uniformly bounded, i.e., $1\leq \lim_{t\to \infty }L<\infty \Leftrightarrow 0<\lim_{t\to \infty }T\leq T\left(0\right)$.

Let $F≜\int_{0}^{t}{∥z∥}^{2}dt$; we can deduce that $\lim_{t\to \infty }F=\int_{0}^{\infty }{∥z∥}^{2}dt\leq -\frac{T\left(0\right)\int_{0}^{\infty }\dot{V}\left(z,\tilde{v}\right)}{\kappa e^{L}\left(1-\varepsilon_{2}\right)}dt<\infty$. Defining $F=2(z_{1}{\dot{z}}_{1}++z_{n}{\dot{z}}_{n})$, $\dot{z}$ is uniformly bounded with *F*. According to Lemma 5, we can obtain that $\lim_{t\to \infty }\dot{F}=0\Leftrightarrow \lim_{t\to \infty }z=0\Leftrightarrow \lim_{t\to \infty }x=0\Leftrightarrow \lim_{t\to \infty }q_{ev}=0$ and $\lim_{t\to \infty }\omega_{e}=0$. This can prove that the proposed update method for dynamic predictive control is locally stable and converges. Hence, the proof of Theorem 1 is completed. □

**Remark** **1.** *The control laws designed for the unknown terms that the system is subjected to are denoted by ${\hat{v}}_{1}$ and ${\hat{v}}_{2}$ in (22) in this study.*

Step 2. Design of a control law for *u*.

Based on the sliding mode variable *s* and the dynamic prediction form of the control torque defined in (23), the control law for spacecraft control torque is defined as follows:

(32) $$\begin{array}{cc} u & =u_{1}+u_{2},\\ u_{1} & =J_{0}\frac{4e^{-L_{2}}I_{3}}{T_{c2}}\frac{|\omega_{e}|\chi -a}{{\left|\vartheta \right|}^{\frac{1}{2}}+\left|\omega_{e}\right|}-J_{0}\chi +\omega^{\times }J_{0}\omega +J_{0}{\hat{v}}_{2},\\ u_{2} & =k_{0}sgn\left(s\right),\\ \chi & =\frac{C_{v}}{T_{c2}}{\left(\alpha {\left|s\right|}^{p}+{\beta |s|}^{q}\right)}^{k}sgn\left(s\right),\\ a & =\frac{C_{v}^{2}}{2T_{c2}}\left(\alpha p\right|q_{ev}{|}^{p-1}+\beta q|q_{ev}{|}^{q-1})\omega_{e},\\ k_{0} & =b_{0}+b_{1}∥\omega ∥+b_{2}{∥\omega ∥}^{2}, \end{array}$$

where $T_{c2}$ is the predefined-time constant for system stability, and $b_{0}$, $b_{1}$, and $b_{2}$ are predefined positive constants that satisfy the definition in (6).

**Theorem** **2.** *Consider the spacecraft described by (1) and (2) and assume that the initial relative attitude does not contain singularities. If the control torque is given by (23) and (32), then the origin of the attitude tracking error system described by (4) and (5) is predefined time stable.*

**Proof.**  Taking the time derivative of the proposed sliding mode variable *s*:

(33) $$\begin{array}{c} \dot{s}={\dot{\omega }}_{e}+\frac{|\omega_{e}|{\dot{\omega }}_{e}}{{\left|\vartheta \right|}^{\frac{1}{2}}}+\frac{\frac{C_{v}^{2}}{2T_{c1}}\left(\alpha p\right|q_{ev}{|}^{p-1}+\beta q|q_{ev}{|}^{q-1})\omega_{e}}{{\left|\vartheta \right|}^{\frac{1}{2}}} \end{array}$$

Substituting the dynamic model of the system (5) into (33), we obtain:

(34) $$\begin{array}{cc} \dot{s} & =\omega_{e}^{\times }C_{e}\omega_{d}-C_{e}{\dot{\omega }}_{d}-J_{0}^{-1}\omega^{\times }J_{0}\omega +J_{0}^{-1}u+J_{0}^{-1}d\\ \; & +\frac{|\omega_{e}|\left(\omega_{e}^{\times }C_{e}\omega_{d}-C_{e}{\dot{\omega }}_{d}-J_{0}^{-1}\omega^{\times }J_{0}\omega +J_{0}^{-1}u+J_{0}^{-1}d\right)}{{\left|\vartheta \right|}^{\frac{1}{2}}}\\ \; & +\frac{\frac{C_{v}^{2}}{2T_{c1}}\left(\alpha p\right|q_{ev}{|}^{p-1}+\beta q|q_{ev}{|}^{q-1})\omega_{e}}{{\left|\vartheta \right|}^{\frac{1}{2}}} \end{array}$$

Substituting (34) into (32) gives:

(35) $$\begin{array}{cc} \dot{s} & =\frac{4e^{-L_{2}}}{T_{c2}}\frac{|\omega_{e}|\chi -a}{{\left|\vartheta \right|}^{\frac{1}{2}}+\left|\omega_{e}\right|}-\chi -J_{0}^{-1}k_{0}sgn\left(s\right)+J_{0}^{-1}d\\ \; & +\frac{|\omega_{e}|\left(\frac{4e^{-L_{2}}\left|\omega_{e}\right|\chi^{-a}}{T_{c2}}\frac{1}{{\left|\vartheta \right|}^{\frac{1}{2}}+\left|\omega_{e}\right|}-\chi -J_{0}^{-1}k_{0}sgn\left(s\right)+J_{0}^{-1}d\right)}{{\left|\vartheta \right|}^{\frac{1}{2}}}\\ \; & +\frac{\frac{C_{v}^{2}}{2T_{c1}}\left(\alpha p\right|\omega_{e}{|}^{p-1}+\beta q|\omega_{e}{|}^{q-1})\omega_{e}}{{\left|\vartheta \right|}^{\frac{1}{2}}} \end{array}$$

Based on the definition in (6), there exists $k_{0}=b_{0}+b_{1}∥\omega ∥+b_{2}{∥\omega ∥}^{2}\geq ∥d∥$, and we can derive from (35) that:

(36) $$\begin{array}{cc} \dot{s} & =-\chi -J_{0}^{-1}k_{0}sgn\left(s\right)+J_{0}^{-1}d+\frac{|\omega_{e}|\left(-J_{0}^{-1}k_{0}sgn\left(s\right)+J_{0}^{-1}d\right)}{{\left|\vartheta \right|}^{\frac{1}{2}}}\\ \; & \leq -\chi =-\frac{C_{v}}{T_{c2}}{\left({\alpha |s|}^{p}+{\beta |s|}^{q}\right)}^{k}sgn\left(s\right) \end{array}$$

Consider the following Lyapunov function candidate $V_{3}=\left|s\right|$. Taking the time derivative of $V_{3}$ yields:

(37) $$\begin{array}{c} {\dot{V}}_{3}=\dot{s}sgn\left(s\right)\leq -\frac{C_{v}}{T_{c2}}{\left({\alpha |s|}^{p}+{\beta |s|}^{q}\right)}^{k}=-\frac{C_{v}}{T_{c2}}{\left(\alpha V_{3}^{p}+\beta V_{3}^{q}\right)}^{k} \end{array}$$

According to Lemma 1, in the convergence phase, the system is predefined time stable, and the predefined time is $T_{c2}$.

Once the tracking trajectory of the system undergoes a phase of stabilization converging to the origin, i.e., s=0 when $t>T_{c2}$, by defining the variables, we can obtain:

(38) $$\begin{array}{c} \omega_{e}=-\frac{C_{v}}{T_{c1}}\left(\alpha \right|q_{ev}{|}^{p}+\beta |q_{ev}{{|}^{q})}^{k}sgn\left(q_{ev}\right) \end{array}$$

where $T_{c1}$ is also the predefined-time constant for system stability.

Consider the following Lyapunov function candidate $V_{4}=\left|q_{ev}\right|$. Taking the time derivative of $V_{4}$ yields:

(39) $$\begin{array}{c} {\dot{V}}_{4}={\dot{q}}_{ev}sgn\left(q_{ev}\right)\leq -\frac{C_{v}}{T_{c1}}\left(\alpha \right|q_{e\nu }{|}^{p}+\beta |q_{e\nu }{{|}^{q})}^{k}=-\frac{C_{v}}{T_{c1}}{\left(\alpha V_{4}^{p}+\beta V_{4}^{q}\right)}^{k} \end{array}$$

Similarly, according to Lemma 1, the system converges stably to the origin in the predefined time $T_{c2}$. Moreover, the system under the control of (23) and (32) converges stably to the origin within the predefined-time constant $T_{c}=T_{c1}+T_{c2}$. Hence, the proof of Theorem 2 is completed. □

Therefore, from Theorems 1 and 2, we conclude that $q_{ev}$ and $\omega_{e}$ converges to the origin within the predefined time.

**Remark** **2.** *The controller designed in this paper relaxes the torque constraint required by general dynamic prediction techniques. Moreover, the upper bound of the predefined-time constant for the closed-loop system in the results is independent of the initial conditions, making the parameter selection of the controller more convenient.*

**Remark** **3.** *Considering that the sign function sgn(s) will lead to system chattering, the sgn(s) in the proposed controller (32) is modified to the following form:*

(40) $$\begin{array}{c} sgn\left(s\right)=\left\{\begin{array}{c} \frac{s}{|s|+0.01},\left|s\right|>\varepsilon \\ \varepsilon I_{3},\left|s\right|\leq \varepsilon \end{array}\right. \end{array}$$

*where ε∈R is a small positive constant.*

**Remark** **4.** *The derivatives of the Lyapunov function in this study are scaled exclusively in the presence of system uncertainty. In contrast, other control schemes undergo multiple scaling steps in their derivation process. As a result, the proposed upper bound on the convergence time presented in this paper is notably less conservative.*

## 5. Simulation Results

In this section, we validate the efficacy of the proposed control scheme through numerical simulation. The nominal inertia matrix $J_{0}$ of the spacecraft is defined with the following parameters: J=[21,1.4,0.8;1.4,18,1.2;0.8,1.2,17] kg· m^2^. In the simulation, 30% of the inertia matrix is given as a deviation, which is represented as the uncertain part of the inertia matrix $\Delta J=0.3J_{0}$. The spacecraft is equipped with MWs drive, which is a rotating body fixed inside the spacecraft, with $J_{m}=diag\left(0.03,0.08,0.08\right)$ kg· m^2^. The reference attitude is $q_{d}=col\left(\sin\left(t\right),\cos\left(t\right),1,0\right)$. The control parameters are chosen as k=0.5, p=1, q=3, α=3, β=3, $T_{c}=10$, $b_{0}=2.01$, $b_{1}=16.2$, $b_{2}=1.9$, $\rho_{1}=1.2$, and $\rho_{2}=1.54$. The saturation problem of the actuator has been considered during the system simulation, and the torque of the actuator is limit to [−5,5] N· m.

**Remark** **5.** *The control parameters in the proposed predefined-time control strategy are critical for achieving optimal performance. Gain parameters (k, α, β) influence the convergence speed and robustness of the control system. Typically, higher values of α and β enhance the system’s ability to reject disturbances and handle uncertainties, but they may also increase control effort and cause chattering. The parameter k should be chosen to balance fast convergence and system stability. Time constants ($T_{c}$, $T_{c1}$, and $T_{c2}$) define the predefined stabilization time. Smaller values lead to faster convergence but require more precise control actions. A moderate starting value is recommended, which can then be fine-tuned based on the desired system performance and response. Disturbance parameters ($b_{0}$, $b_{1}$, and $b_{2}$) estimate the upper bounds of disturbances and uncertainties. Accurate estimation is crucial for maintaining robustness. Initial values can be based on prior system knowledge and adjusted according to observed disturbance levels during operation. Adaptive parameters ($\rho_{1}$, $\rho_{2}$) control the adaptation rate of the dynamic predictive control scheme. Higher values result in quicker adaptation to changing conditions but may risk instability if set too high. By adjusting the corresponding control parameters, optimal performance can be achieved under different spacecraft configurations and mission requirements.*

**Figure 3:**
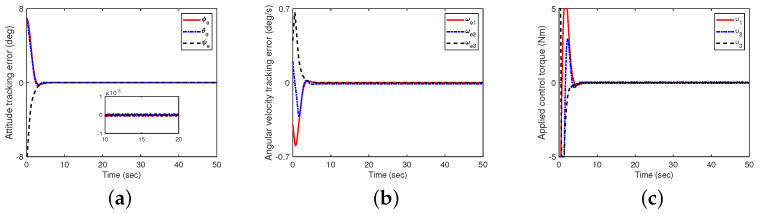
The impact of controllers (23) and (32) on attitude tracking (with uncertain factors, no disturbances, and no measurement noise). (**a**) Attitude tracking errors in Euler angles with a 3−1−2 rotation sequence; (**b**) angular velocity tracking errors; (**c**) control torque.

First, it is assumed that there are no disturbance and measurement noises (define this as Case A). In this case, the initial conditions of the system are q(0)=col(0.4,−0.2,0.65,0.6) and ω(0)=col(0.5,0.4,0.3) deg/s. The simulation results are shown in Figure 3. It can be observed that the attitude tracking errors of the spacecraft in Euler angles with a 3−1−2 rotation sequence (i.e., $\phi_{e}$, $\theta_{e}$ and $\psi_{e}$) are less than 0.002 degrees when t≥10s, which indicates that the proposed control scheme in this paper can achieve perfect spacecraft attitude tracking.

**Figure 4:**
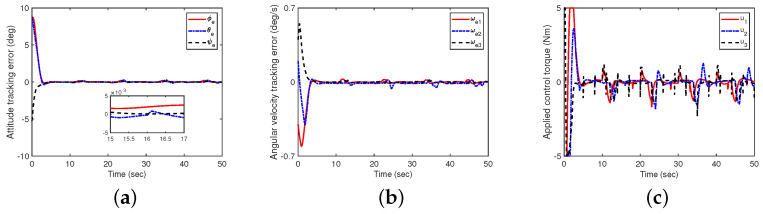
The impact of controllers (23) and (32) on attitude tracking (with uncertainties and disturbances, without measurement noise). (**a**) Attitude tracking errors in Euler angles with a 3−1−2 rotation sequence; (**b**) angular velocity tracking errors; (**c**) control torque.

Next, the case with disturbances is addressed (define this as Case B). In this case, the elements of the initial attitude q(0)=col(−0.3,−0.1,0.5,0.7) and initial angular velocity ω(0)=col(−0.4,0.2,0.4) deg/s are given, respectively. To investigate the robustness performance of the closed-loop system, an external disturbance $u_{d}=0.1col(\sin0.5t,\cos0.5t$, sin0.4t) N· m is given. The results are shown in Figure 4, indicating that the attitude tracking errors of in Euler angles are less than 0.008 degree at the steady-state stage.

**Figure 5:**
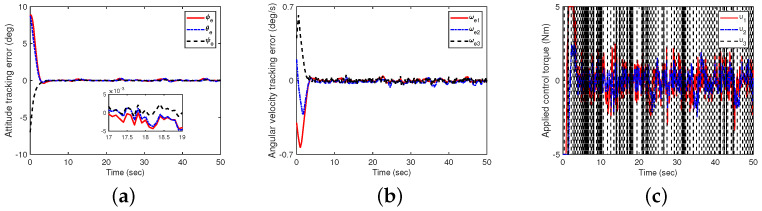
The impact of controllers (23) and (32) on attitude tracking in the presence of uncertainty, disturbances, and measurement noise. (**a**) Attitude tracking errors in Euler angles with a 3−1−2 rotation sequence; (**b**) angular velocity tracking errors; (**c**) control torque.

Furthermore, we discuss the case where measurement noise is present (define this as Case C). In this case, the initial values of the attitude q(0) and angular velocity ω(0) are randomly selected from the range [−0.4,0.8] and [−0.5,0.5] deg/s, respectively. The measurement noise signals defined in the controllers during the simulation calculations are given according to An-Min Zou et al.’s method [37]. The results in Figure 5 show that at the steady state, the attitude tracking errors in the Euler angles are less than 0.01 degrees. The excellent performance of the proposed control scheme is further evidenced by the root mean square error (RMES) of the attitude tracking presented in Table 1.

Finally, a comprehensive comparison is conducted between the proposed predefined-time dynamic predictive controller, traditional PID control (as shown in (41)), and the existing fixed-time control (as shown in (42)) methods under the same conditions:

(41) $$\begin{array}{c} u_{\mathrm{PID}}=K_{p}e+K_{i}\int e\;dt+K_{d}\dot{e} \end{array}$$

where $K_{p}$, $K_{i}$, and $K_{d}$ are the proportional, integral, and derivative gains, respectively. The PID controller is widely used due to its simplicity and effectiveness, but it has significant limitations when dealing with uncertainties, bounded actuators, and external disturbances:

(42) $$\begin{array}{c} u=-\left[\left(k_{2}\gamma +k\right){\mathrm{sig}}^{\alpha }\left(\xi \right)+\left(k_{3}\gamma +k\right){\mathrm{sig}}^{\beta -1+\alpha_{1}}\left(\xi \right)\right]-f\left(\omega ,\omega_{d},{\dot{\omega }}_{d}\right)-\kappa \beta J\mathrm{diag}(|q_{e}{|}^{\beta -1}){\dot{q}}_{e} \end{array}$$

where k>0, $k_{i}>0$ (i=2,3) are constants, and $\mathrm{diag}(|q_{e}{|}^{\beta -1})$ is a diagonal matrix. This fixed-time method provides a guaranteed convergence time but faces challenges in parameter tuning and robustness under high levels of uncertainties and disturbances.

**Figure 6:**
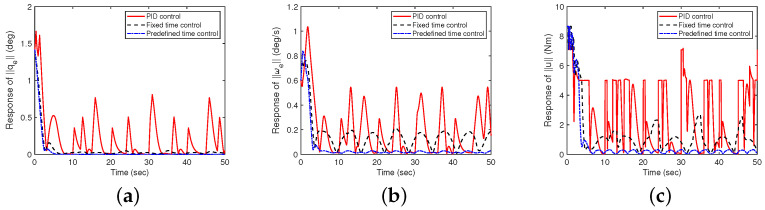
Performance comparison between the controllers designed in this paper and the fixed-time controller (with uncertainties and measurement noises). (**a**) Response of $∥q_{e}∥$; (**b**) response of $∥\omega_{e}∥$; (**c**) response of $∥u∥$.

As shown in Figure 6, even with simultaneous uncertain factors and measurement noise affecting the spacecraft system, the proposed predefined-time dynamic predictive method exhibits superior performance in terms of spacecraft attitude angle tracking, angular velocity response, and control torque jitter compared to both PID and fixed-time controllers. The PID controller shows significant performance degradation under uncertainties and disturbances. The fixed-time controller offers improvements but still lacks the flexibility and robustness of the proposed method.

In conclusion, the proposed control strategy demonstrates strong robustness to existing uncertainties and disturbances, achieving the control goal of attitude tracking more effectively than traditional PID and fixed-time control methods. This highlights the practical benefits of the proposed method for advanced spacecraft attitude control applications.

## 6. Conclusions

This paper presents a novel predefined-time control scheme for rigid spacecraft, leveraging dynamic predictive techniques to address the robustness challenges posed by system uncertainties and environmental disturbances. The proposed approach combines the strengths of quaternion-based attitude representation and predefined-time control, ensuring high precision and robustness against external perturbations. Key contributions include the introduction of adaptive global optimization for period updates, easing parameter adjustments for predefined-time control, and developing a continuous and robust control law using terminal sliding mode control and predictive methods. Our method significantly surpasses traditional control strategies by providing rapid convergence to desired attitudes with exceptional accuracy as evidenced by simulation results showing attitude tracking errors reduced to below 0.01 degrees. The control strategy’s flexibility allows for adaptations to different spacecraft models, enhancing its applicability in complex space missions. Furthermore, the robustness of the control scheme against disturbances and its capacity to handle uncertainties without performance degradation highlight its practical benefits for future aerospace applications. Future work will focus on expanding the applicability of this strategy to more complex models and testing its effectiveness in real-world space mission scenarios. This approach, due to its robust and adaptable nature, also has potential applications in other areas requiring precise dynamic control, such as robotics and unmanned aerial vehicles.

## Figures and Tables

**Table 1:** Attitude tracking root mean square error (RMES).

Euler Angles	Case A	Case B	Case C
$\phi_{e}$ (deg)	0.129	0.132	0.348
$\theta_{e}$ (deg)	0.110	0.110	0.1438
$\psi_{e}$ (deg)	0.069	0.067	0.090

## Data Availability

The raw data supporting the conclusions of this article will be made available by the authors on request.
